# The Effect of Percutaneous Coronary Intervention on QT Dispersion and the Association Between Them: A Systematic Review

**DOI:** 10.7759/cureus.36226

**Published:** 2023-03-16

**Authors:** Mahdi Dahrab, Sai Pranathi Gaddipati, Keval B Patel, Tirath Patel, Ashwith R Gaddam, Manisha Jain, Thulasi Ram Gudi, Dakshin Meenashi Sundaram, Kamran Mahfooz, Advait M Vasavada

**Affiliations:** 1 Internal Medicine, McMaster University, Hamilton, CAN; 2 Internal Medicine, Mallareddy Medical College for Women, Hyderabad, IND; 3 Surgery, Narendra Modi Medical College, Ahmedabad, IND; 4 Surgery, American University of Antigua, St. John, ATG; 5 General Medicine, Emilio Aguinaldo College, Manila, PHL; 6 Internal Medicine, Shri Bhausaheb Hire Government Medical College, Dhule, IND; 7 Internal Medicine, Merit Health River Region, Vicksburg, USA; 8 Internal Medicine, Employees’ State Insurance Corporation (ESIC) Medical College and Post Graduate Institute of Medical Science and Research (PGIMSR), Chennai, IND; 9 Internal Medicine, Lincoln Medical Center, New York, USA; 10 Internal Medicine, Meghji Pethraj (MP) Shah Medical College, Jamnagar, IND

**Keywords:** ecg abnormalities, corrected qt interval, angioplasty and stenting, qt dispersion, primary percutaneous coronary intervention (pci)

## Abstract

Electrocardiography (ECG) parameters are significant in the prognosis of ischemia and other cardiovascular conditions. Reperfusion or revascularization techniques are essential in reestablishing blood flow to ischemic tissues. This study aims to demonstrate the association between percutaneous coronary intervention (PCI), a revascularization technique, and the electrocardiography (ECG) parameter, QT dispersion (QTd). We conducted a systematic review of the association between PCI and QTd through a literature search in three electronic databases, ScienceDirect, PubMed, and Google Scholar, for empirical studies published in English. Review Manager (RevMan) 5.4 (Cochrane Collaboration, Oxford, England) was used for statistical analysis. Of 3,626 studies, 12 articles met the inclusion criteria, enrolling a total of 1,239 patients. After a successful PCI procedure, QTd and corrected QT (QTc) tremendously reduced at various time intervals with statistical significance in most of the studies. There was a clear association between ECG parameters QTd, QTc, and corrected QT dispersion (QTcd), and PCI, in that there is a considerable reduction in these ECG parameters after PCI treatment.

## Introduction and background

Cardiovascular diseases such as coronary heart disease (CHD), ischemic heart disease (IHD), and acute myocardial infarction (AMI) are leading causes of death across the globe. They have been reported to be on the rise, branded by many researchers as a pandemic that knows no borders [1]. The disease is known to affect not only high-income nations but also middle- and low-income countries worldwide, with approximately 80% of global deaths associated with cardiovascular diseases hitting third-world and developing countries [2]. Besides, it is estimated that nearly 30% of these fatalities are attributed to the prevalence of cardiovascular diseases in these countries [3]. Several studies have revealed that about 1.8 million individuals develop AMI annually in the United States, with a 90% chance of developing thrombosis among patients with ST-elevation myocardial infarction (STEMI) [4]. As such, it is clear that reperfusion can be employed as an ideal interventional treatment for individuals suffering from STEMI [5].

The reperfusion treatment modalities widely used include fibrinolysis (comprising of fibrinolytic treatment drugs such as streptokinase) and primary percutaneous coronary intervention (PCI), commonly referred to as coronary angioplasty or just angioplasty [6]. PCI is an interventional procedure employed in treating angina and myocardial infarction characterized by narrowed coronary arteries. As a result, PCI is sometimes utilized as an emergency intervention to help patients with heart attacks [7]. Therefore, the two treatment methods are the most preferred since they can restore blood flow to the heart and ischemic tissues [6]. In most cases, PCI is performed within the first 1-2 hours of the occurrence of the disease. By doing so, PCI aims to offer quicker and more adequate blood supply to the ischemic vessels and tissues. On the other hand, when applied at an ideal time and in a proper manner, fibrinolytic therapies are instrumental in lowering the high rates of hospital morbidity and mortality among patients [8].

The diagnosis of coronary artery disease (CAD) has been mostly done under the 12-lead electrocardiogram (ECG), which is by far a noninvasive procedure with other benefits such as determining and estimating the size and localization of myocardial ischemia in addition to CAD diagnosis. It is worth mentioning that ECG and body chest X-ray scans were the most common procedures used in diagnosing cardiac disease [9]. With technological advancements, this has changed and led to incorporating QT intervals on the ECG surface. This form of QT measures the total time the ventricles are depolarized and repolarized. The QT interval variations in leads conforming to various heart regions represent regional variations in ventricular repolarization resulting in a heterogeneity known as the QT interval dispersion [10]. One study observed that atypically high QT dispersion (QTd) was articulated to an increased chance of dying from an arrhythmic cause in several heart conditions, including CAD [11]. Furthermore, according to reports, a rise in QTd among individuals with IHD can predict the development of fatal ventricular tachyarrhythmias and sudden cardiac death [12].

Furthermore, in patients with known CAD, QTd was shown to be considerably increased during an anginal episode compared to painless circumstances [13], while in acute STEMI, prolonged corrected QT (QTc) dispersion (QTcd) was linked to the severity of the CAD [14]. These findings further indicate that QTd can be increased by ischemia. Thus, since PCI has been widely adopted and performed to manage ischemia in CAD patients, it can be seen that, as a consequence, PCI can be significant in reducing QTd. Moreover, another research demonstrated that after successful coronary artery revascularization, QT interval dispersion reduced, while it rose with restenosis [15]. Therefore, the implication of such results can be used to indicate that QTd may serve as a prognostic indicator after performing PCI. In this light, this current study was conducted to determine the association between QT and PCI. In addition, the study also aims to determine the effect of PCI on QTd among patients with cardiovascular diseases.

## Review

Materials and methods

Study Design and Literature Search

The current study is a systematic review of 12 primary research studies done following the guidelines and principles contained in the Preferred Reporting Items for Systematic Reviews and Meta-Analyses (PRISMA) framework [16]. A systematic literature search was comprehensively conducted across three electronic databases, ScienceDirect, PubMed, and Google Scholar, for English published articles exploring the association between PCI and QTd.

Search Strategy

For an effective and efficient literature search, the study utilized a set of keywords and incorporated the Boolean expressions "OR" and "AND." Therefore, the search criteria included the following initial keywords: "PCI," OR "PPCI," OR "primary percutaneous coronary intervention," AND "QT interval dispersion," OR "QT dispersion" OR "QTd" AND "QT correction," OR "QTc," AND "angioplasty," OR "coronary angioplasty," OR "percutaneous transluminal coronary angioplasty," OR "PTCA." The search was restricted to full-text articles published between 2000 and 2023 in English. To uncover more pertinent manuscripts, references from the original recognized papers and reviews were looked through.

Inclusion Criteria

To carefully select pertinent articles for this study's analysis, the following inclusion criteria were used to narrow down the pool of sources acquired. Primary articles exploring the association between QT and PCI were included. Empirical English published articles between 2009 and 2023 were included to avoid the omission of misinterpretations during translations from one language to English. Studies involving PCI and QTd in human subjects were included.

Exclusion Criteria

Studies were excluded based on the following criteria: secondary materials such as magazines, abstracts, case reports, other reviews, and newspaper articles exploring QT and PCI were excluded. Primary studies evaluating QT and PCI in animal subjects were excluded. Studies conducted in other languages apart from English and prior to 2009 were also excluded.

Data Extraction and Quality Appraisal

Two independent reviewers selected and extracted data from studies that complied with the Population, Intervention, Comparison, Outcomes, and Study (PICOS) framework's inclusion criteria [17]. These reviewers obtained information on the authors, study procedures, participant characteristics, intervention, comparison, and significant results or outcomes, among other things. To aid in the resolution of disputes for the extraction of relevant data, a second reviewer was engaged. The included studies' quality was evaluated using the Newcastle-Ottawa Scale (NOS). The NOS scoring system assessed the categories of research participant selection, result comparability, and outcome quality [18].

Results

**Figure 1:**
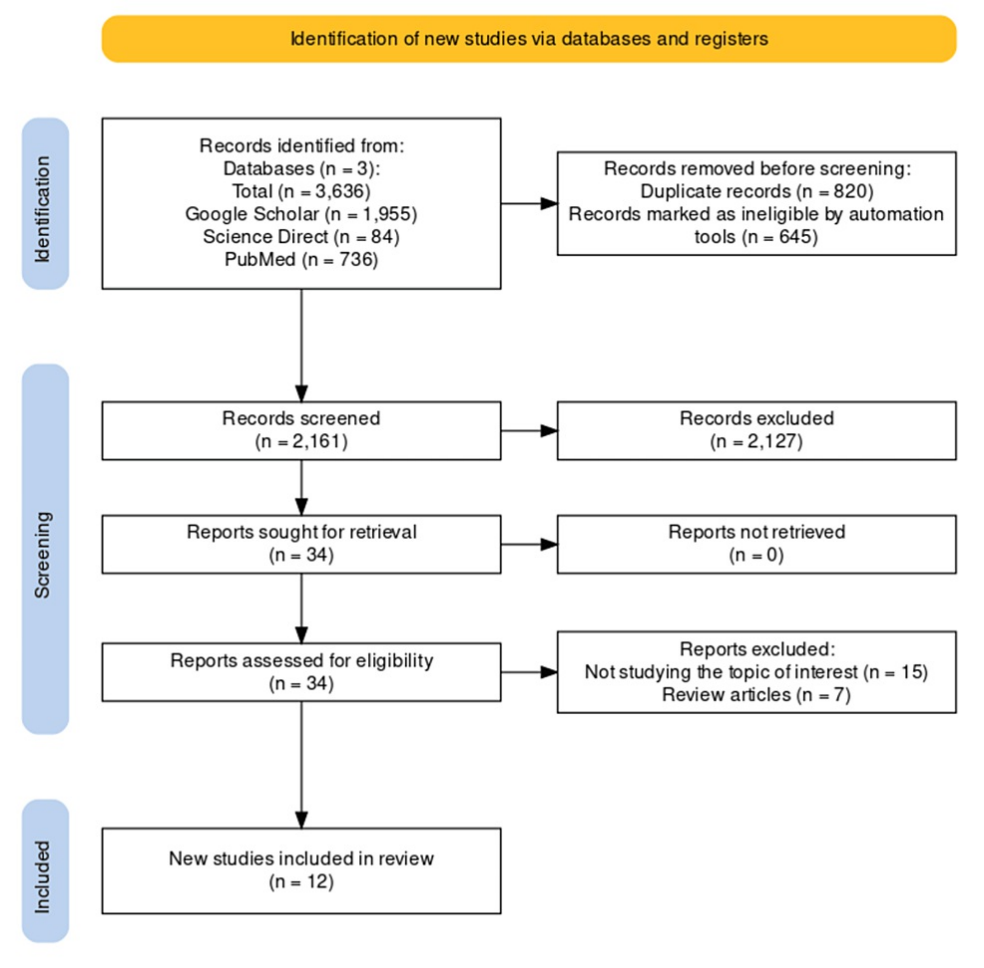
PRISMA Flowchart PRISMA: Preferred Reporting Items for Systematic Reviews and Meta-Analyses

A total of 3,626 articles were identified from various databases and from scanning through their reference lists. Only 12 of these papers met the criteria for inclusion and were retrieved for analysis. All of the articles investigated the relationship between QT and PCI. The PRISMA chart is shown in Figure 1.

Study Characteristics

All 12 studies were analyzed, and the extracted data characteristics are shown in Table 1.

**Table 1:** Study Characteristics PCI: percutaneous coronary intervention, STEMI: ST-elevation myocardial infarction, PIA: postinfarction angina, QTd: QT dispersion, QTc: corrected QT, STR: ST‐segment resolution, Tp-Te: T‐peak‐to‐T‐end interval, PTCA: percutaneous transluminal coronary angioplasty, HRV: heart rate variability, AMI: acute myocardial infarction, TPE: T-wave peak to end, CABG: coronary artery bypass graft, SK: streptokinase [19-30]

Study	Interventions	Objective	Sample size (number)	Follow-up	Primary outcomes	Results
Ahmed et al., 2018 [19]	PCI	To study the difference in acute electrocardiographic findings between STEMI patients with and without PIA and to assess in‐hospital arrhythmias in both groups	Total=238 (PIA=42, no PIA=196)	Pre- and immediately post-procedure	STR at 90 minutes, QTc and QTd, Tp‐Te, and dispersion and Tp‐Te/QT ratio	Patients with PIA had higher rates of STR (p<0.0001), while patients without PIA had higher values of QTc (p=0.006), QTd (p=0.001), Tp‐Te interval (p=0.001), Tp‐Te dispersion (p<0.0001), and Tp‐Te/QT ratio (p=0.01) compared to those with angina preceding their incident infarction (PIA).
Alasti et al., 2010 [20]	PCI	To evaluate the effect of PCI on surface electrocardiography's depolarization and repolarization parameters in patients with chronic stable angina	Total=96 (male=67, female=29; age=53.2±9.5)	Pre- and 24 hours post-procedure	QT and QTc, JT and corrected JT	There was a significant reduction in QTd and corrected JT dispersion in patients undergoing PCI.
Alici et al., 2013 [21]	Primary PCI	To analyze the additional effect of thrombectomy on QT parameters in patients with STEMI undergoing primary PCI	Total=80 (male=71, female=9; primary PCI group=40, primary PCI+thrombectomy group=40)	Pre- and 24 hours post-procedure	QTd, QTc	At 24 hours, in the thrombectomy group, QTd and QTc were significantly lower in those undergoing primary PCI.
Al Alwany, 2022 [22]	PCI	In patients with chronic total occlusion, to analyze the variations in the QTc dispersion before and after PCI	Total=110 (male=93, female=17; age=57.0±9.1)	Pre- and post-intervention procedure	QTc interval and QTc dispersion	The average QTc interval and QTc dispersion changed significantly before and after PCI.
Aydinlar et al., 2009 [23]	PTCA	In patients undergoing elective PTCA, to analyze parameters of HRV and QTd	Total=26 (male=16, female=10, age=58.3±17.1)	Pre- and immediately post-procedure	HRV and QTd	QTc dispersion was significantly decreased after PTCA, and high-frequency HRV was significantly higher after PTCA, whereas low-frequency HRV and the ratio of low frequency:high frequency (3.3±1.9 versus 2.1±1.2) were significantly decreased after PTCA.
Babapour et al., 2018 [24]	Primary PCI on ventricular repolarization	To assess the impact of primary PCI on ventricular repolarization in patients with AMI by measuring QTd	Total=77 (male=60, female=17)	QT pre- and post-24 hours	QTd	A drop in QTd was seen 24 hours after PCI. However, it was not very noticeable.
Eshraghi et al., 2017 [25]	PCI and morphine	In patients undergoing primary PCI, to analyze the additional effect of morphine post-conditioning on the QTd on the anterior descending cardiac artery	Total=77 (male=61, female=16; control=31, morphine consumption before PCI=46)	24 hours post-procedure	QTd	Morphine consumption before PCI can further reduce QTd value in an electrocardiogram for PCI as compared to patients who did not take morphine before PCI.
Eslami et al., 2013 [26]	PCI	In STEMI patients, to analyze the effect of PCI in QTc, QTd, and TPE	Total=80 (male=60, female=20)	Pre- and 24 hours post-procedure	QTc, QTd, and TPE	A significant reduction was observed in QTd and TPE following PCI.
Hassan et al., 2018 [27]	Primary PCI plus thrombectomy	To evaluate the effects of primary PCI combined with thrombectomy versus primary PCI alone on QTd in patients with acute STEMI	Total=48 (male=33, female=15)	Before and after 24 hours of the procedure	QTc and QTd	After 24 hours, primary PCI decreased QTc and QTd, and thrombectomy or no thrombectomy had no discernible influence on these parameters.
Mirbolouk et al., 2014 [28]	PCI and CABG	In chronic ischemia, to analyze and compare the effect of PCI and CABG on QT parameters	Total=141 (PCI=70, CABG=71)	Pre-, immediately, 24 hours post-procedure, and seven days post-procedure	QTc dispersion and QTc interval	PCI and CABG can improve QT parameters in chronic ischemia.
Pan et al., 2011 [29]	Primary PCI	To analyze the effect of PCI in corrected QTd before and after successful PCI	Total=81	Pre- and 24 hours post-procedure	QTc dispersion	QTc dispersion was significantly shorter after successful PCI.
Valizadeh et al., 2020 [30]	SK versus intervention (PCI)	To analyze the impact of SK and PCI before and after treatment in patients with STEMI	Total=185 (SK=115, PCI=70; male=137, female=48)	Pre- and 24 hours post-procedure	QTd and QTc	QTd significantly decreased in the primary PCI group (p=0.022).

Quality Assessment

The NOS scoring system as mentioned above in the methods was used to critically appraise the studies. A detailed account of the quality assessment done is shown in Table 2.

**Table 2:** Quality Appraisal [19-30]

Study	Selection (maximum: 4)	Comparability (maximum: 1)	Outcome (maximum: 3)	Total score	Quality
Ahmed et al., 2018 [19]	2	1	2	5	Moderate
Alasti et al., 2010 [20]	3	1	1	4	Moderate
Alici et al., 2013 [21]	2	1	3	6	Moderate
Al Alwany, 2022 [22]	3	1	2	6	Moderate
Aydinlar et al., 2009 [23]	3	1	3	7	High
Babapour et al., 2018 [24]	3	1	2	6	Moderate
Eshraghi et al., 2017 [25]	2	1	2	5	Moderate
Eslami et al., 2013 [26]	2	1	3	6	Moderate
Hassan et al., 2018 [27]	3	1	3	7	High
Mirbolouk et al., 2014 [28]	3	1	2	6	Moderate
Pan et al., 2011 [29]	3	1	3	7	High
Valizadeh et al., 2020 [30]	2	1	2	5	Moderate

Discussion

Studying QT dispersion in PCI is important for several reasons. First, PCI can cause changes in the QT interval, which may increase the risk of arrhythmias. Second, patients undergoing PCI may have preexisting rhythm abnormalities, which can affect the success of the procedure and the patient's prognosis. By studying QT dispersion, clinicians can better understand the risks associated with PCI and develop strategies to minimize these risks. Additionally, QT dispersion can be used to monitor patients after PCI to assess their risk of arrhythmias and guide the management of any arrhythmias that may occur. This can help improve patient outcomes and reduce the risk of complications by noninvasively detecting irregularities [10].

The objective of the current review is to evaluate the association between QT dispersion and percutaneous coronary intervention. In doing so, we further evaluated the impact of PCI in the reduction of QTd by comparing baseline QTd and that at various points after the intervention of PCI. Furthermore, we establish the effectiveness of the PCI procedure versus other treatments, including thrombectomy [21,27], CABG [28], and streptokinase [30], in effecting QTd. Results from most of the studies indicate that PCI is associated with QTd, by which performing PCI procedures significantly reduces QTd, QTc, and QTcd. In most studies, immediately after PCI intervention and as time goes on (two hours, 24 hours, and 2-7 days post-PCI), QTd reduces significant differences between pre-PCI and post-PCI periods. Similarly, a substantial decrease in QTc after 24 hours of PCI intervention was present between the pre- and post-PCI periods. However, despite showing a reduction in QTd in the PCI group, this finding should be explored in future studies.

Numerous research has drawn links between QTd and arrhythmic events among patients with heart failure, post-MI, CHD, and hypertrophic cardiomyopathy [31]. According to Karagounis et al. [32], this association has mainly been observed to correlate between QTd positively and left ventricular ejection fraction in individuals with MI [32]. Our study demonstrates that PCI is closely associated with QTd, which can be articulated by the fact that QTd is affected by ischemic heart conditions (ischemia). In this light, further findings have supported the idea that ischemia can lengthen QTd [14]. Therefore, it can be observed that as a result of PCI's widespread adoption and use in attempts to treat ischemia in CAD patients, PCI can significantly shorten QTd [33]. Previous literature has reported a substantial decrease in QTd after effective coronary revascularization or thrombolysis [34,35]. According to Pan et al. [29], one of the explanations for the reduction of arrhythmic events in patients receiving PCI treatment may be articulated as the QTd shortening and subsequent decrease in ventricular arrhythmia inducibility. The findings of Pan et al. [29] further demonstrate the discovery of major adverse cardiac events, mainly associated with patients with STEMI, to be substantially attributed to the absolute corrected QTd change following PCI.

Similarly, another research study points out that early reduction in QTd following a primary PCI treatment is closely associated with the return of microvascular reperfusion, which provides crucial additional prognostic information in STEMI patients [36]. In addition, another study established that a considerable shortening of QTd can serve as an additional electrocardiographic marker for effective reperfusion in patients with MI [37]. According to the findings of the community-based Strong Heart Study, consequently, computerized QTd measurements have been suggested as a tool for noninvasive risk stratification of patients who are more likely to die from cardiovascular causes, while the prolongation of the corrected QTd after hemodialysis has been found to predict cardiovascular death in hemodialysis patients [38,39].

Although the patterns of changes in study parameters, such as QTd and QTc, were relatively higher in the PCI group and were visible soon after procedures, these changes should be evaluated further by pooling all the data together [28]. Of note, Cagli et al. [40] reported an increase in QTd 24 hours post-procedures. Nonetheless, our analysis is consistent and similar to the results obtained by Valizadeh et al. [30], who observed that the QTc and QTcd in the PCI group showed a declining pattern right after surgery. However, a significant reduction did not happen until 24 hours and seven days posttreatment. Yunus et al. [15] demonstrated a significant decrease in QTd 24 hours after PCI in 37 patients without a history of MI [15]. This can be articulated by the fact that increased sympathetic activity during the first hours of PCI prevented the manifestation of the consequences of ischemia revision [15]. However, the chances of this happening are subtle. As a result, some studies have suggested that QTd variations following effective PCI could serve as independent risk variables to forecast upcoming outcomes [41,42].

Our study established that QTd can be used as a prognostic tool and that there is substantial evidence supporting the findings. This could be interesting as a kickstart point for future prospective studies where all the limitations of our systematic review as delineated below can be addressed and substantial data could be extracted to define a risk stratification tool using QTd with several other parameters [43].

Limitations

The majority of included articles comprised observational studies, some of which employed retrospective data analysis, which may include a bias element. Other inaccuracies may result from unmeasured external confounding factors and the assumption that all QTd measurements in between studies were carried out accurately. This may not have been the case in practice due to human errors during the measuring process. Also, cardiac imaging was not utilized alongside to determine the correlation between QT dispersion and cardiac wall motion abnormalities. Using such a tool could highlight how QTd affects wall motion. Furthermore, various clinical factors, such as hemodialysis, autonomic dysfunction, and electrolyte imbalances in QTd, as well as the effects of cardiologic and non-cardiological drugs, are additional potential confounding factors (not accounted for in our review) that could affect our conclusions.

## Conclusions

Our review demonstrates a clear association between PCI and ECG parameters (QTd, QTc, and QTcd), in that there is a significant reduction in these ECG parameters following the PCI procedure. We also demonstrated that PCI was as effective as other revascularization treatment methods in reducing QTd among patients with various cardiovascular diseases. Therefore, it can be seen that regardless of the technique used, revascularization enhances the electrical activity of the heart. When performed thoroughly, greater benefits can be realized and calculated using ECG parameters. Future prospective studies with a focus to study this phenomenon under controlled measures can be useful, and conclusions could directly affect clinical practice by the inclusion of this parameter in a risk stratification tool.
